# The nucleus accumbens shell: a neural hub at the interface of homeostatic and hedonic feeding

**DOI:** 10.3389/fnins.2024.1437210

**Published:** 2024-07-30

**Authors:** Alina-Măriuca Marinescu, Marie A. Labouesse

**Affiliations:** ^1^Brain, Wire and Behavior Group, Translational Nutritional Biology Laboratory, Department of Health Sciences and Technology, ETH Zurich, Zurich, Switzerland; ^2^Neuroscience Center Zurich, University of Zurich, ETH Zurich, Zurich, Switzerland

**Keywords:** dopamine, food intake, hedonic feeding, homeostatic feeding, neural circuits, nucleus accumbens, nucleus accumbens shell, reward

## Abstract

Feeding behavior is a complex physiological process regulated by the interplay between homeostatic and hedonic feeding circuits. Among the neural structures involved, the nucleus accumbens (NAc) has emerged as a pivotal region at the interface of these two circuits. The NAc comprises distinct subregions and in this review, we focus mainly on the NAc shell (NAcSh). Homeostatic feeding circuits, primarily found in the hypothalamus, ensure the organism’s balance in energy and nutrient requirements. These circuits monitor peripheral signals, such as insulin, leptin, and ghrelin, and modulate satiety and hunger states. The NAcSh receives input from these homeostatic circuits, integrating information regarding the organism’s metabolic needs. Conversely, so-called hedonic feeding circuits involve all other non-hunger and -satiety processes, i.e., the sensory information, associative learning, reward, motivation and pleasure associated with food consumption. The NAcSh is interconnected with hedonics-related structures like the ventral tegmental area and prefrontal cortex and plays a key role in encoding hedonic information related to palatable food seeking or consumption. In sum, the NAcSh acts as a crucial hub in feeding behavior, integrating signals from both homeostatic and hedonic circuits, to facilitate behavioral output via its downstream projections. Moreover, the NAcSh’s involvement extends beyond simple integration, as it directly impacts actions related to food consumption. In this review, we first focus on delineating the inputs targeting the NAcSh; we then present NAcSh output projections to downstream structures. Finally we discuss how the NAcSh regulates feeding behavior and can be seen as a neural hub integrating homeostatic and hedonic feeding signals, via a functionally diverse set of projection neuron subpopulations.

## 1 Introduction

Feeding behavior is a multifaceted physiological process governed by the interaction of homeostatic and hedonic feeding pathways. Homeostatic feeding circuits, primarily located in the hypothalamus, regulate an organism’s energy and nutrient balance by monitoring peripheral signals. These include, for instance, insulin, a pancreatic hormone that modulates blood glucose levels (Rahman et al., 2021), leptin, an adipose-derived anorexigenic hormone (van Swieten et al., 2014), ghrelin, an orexigenic peptide produced in the stomach (Yanagi et al., 2018) or glucagon-like peptide-1 (GLP-1), a gut hormone regulating glucose homeostasis and satiety (Smith et al., 2019). On the other hand, the so-called hedonic feeding circuits involve signals from sensory inputs, learned associations, reward, motivation, and pleasure derived from eating. The nucleus accumbens shell (NAcSh) is one of the key brain regions located at the interface of these two circuits. The NAcSh receives brain-wide inputs from regions deeply involved in both homeostatic and hedonic circuits, making it an ideal intersection point to integrate information about both processes. In turn, the NAcSh sends direct and indirect projections to midbrain structures where it can strongly influence behavioral output related to feeding. In this review, we delineate the general architecture of the NAcSh, starting by outlining inputs to the NAcSh, then presenting its outputs to target structures, focusing primarily on the general organization and cellular architecture. Finally, we discuss the mechanisms by which the NAcSh integrates multi-sourced signals to regulate adaptive feeding behavior. We also discuss the rich diversity of NAcSh output neurons, harboring varied molecular markers, projection circuits or topographical locations. Of note, our review below focuses primarily on the medial NAcSh region, given its predominant roles in feeding behavior as compared to other NAc subregions.

### 1.1 Cellular architecture of the nucleus accumbens shell

The nucleus accumbens (NAc) belongs to limbic circuitries within the ventral forebrain, forming together with the olfactory tubercule (OT) and islands of Calleja the so-called ventral striatum (*VS*). It can be subdivided into several distinct subregions: a core (NAcC), a medial NAcSh and a lateral NAcSh region. 95% of neurons in the NAc consist of medium spiny neurons (MSNs), long-projecting cells that release the inhibitory neurotransmitter γ-Aminobutyric acid (GABA). These include MSNs expressing dopamine 1 receptor (D1R) (D1R-MSNs) and MSNs expressing dopamine 2 receptor (D2R) (D2R-MSNs) [reviewed in Castro and Bruchas (2019)], the two dominant types of dopamine receptors. Dopamine receptors are G-protein coupled receptors (GPCRs) which come in different subtypes. D1R-like receptors (which includes dopamine receptors type 1 and 5: D1R, D5R) are coupled to the G-protein G_s_ and promote cAMP formation by activating adenylyl cyclase, while D2R-like receptors (D2R, D3R, D4R) are coupled to G_i_ and inhibit cAMP formation by inhibiting adenylyl cyclase (Beaulieu and Gainetdinov, 2011). In the NAcSh, MSNs are smaller and appear less spiny as compared to MSNs in the NAcC (Meredith et al., 2008). D3R receptors are also enriched in the NAcSh and predominantly expressed in D1R-MSNs (Schwartz et al., 1998). Of note, it has been shown that MSNs can co-express both D1Rs and D2Rs in the NAcSh in about 17% of MSNs (Bertran-Gonzalez et al., 2008; Beaulieu and Gainetdinov, 2011). The other 5% of remaining striatal cells in the NAcSh are local interneurons (Tepper et al., 2010; Castro and Bruchas, 2019). Interneurons are of three major types: (i) fast-spiking, parvalbumin+ (PV) cells that co-express the endocannabinoid receptor 1 (CB1) (Winters et al., 2012; Schall et al., 2021), (ii) somatostatin+ (SST) cells co-expressing neuropeptide Y (NPY) and nitric oxide synthase (NOS) and lastly, (iii) tonically active cholinergic (CIN) interneurons. As compared to the dorsal striatum (DS) and the NAcC, PV+ interneurons are very sparse in the NAcSh. The distribution of SST+ and CIN+ cells also differs, their numbers being higher in the NAcSh (Castro and Bruchas, 2019). Morphologically, a major difference between the NAcSh and the DS/NAcC is the differential density of matrix and striosome compartments, the two chemical compartments within the striatum characterized by their rich vs. poor density for calcium binding proteins, respectively. The NAcSh contains predominantly calbindin-poor striosomes with few interspersed areas of calbindin-rich matrices, while the opposite is true for the DS and NAcC. Because of this, calbindin-immunoreactivity is commonly used to delineate the NAcSh region (especially its medial section) from the NAcC, as calbindin-immunoreactive cells are nearly absent in the NAcSh (Tripathi et al., 2010). Molecularly, the levels of substance P, calretinin, dopamine (DA), serotonin and norepinephrine receptors are also higher in the NAcSh compared to the NAcC, while enkephalin, calbindin and GABA type A receptor (GABA_A_R) are predominantly present in the NAcC [reviewed in Salgado and Kaplitt (2015)]. Besides the molecular make-up of the NAcSh, inputs and outputs to and from the NAcSh are other major factors differentiating it from other brain regions and influencing its function, which we describe in detail below.

## 2 Inputs to the nucleus accumbens shell

The activity of cells within the NAcSh is regulated by excitatory, inhibitory and neuromodulatory inputs. How do inputs differ across NAc subregions? Two recent studies answered this question by using retrograde tracing techniques to map brain-wide afferents to the entire NAc in a cell-type specific manner. Ma et al. (2020) used cholera-toxin subunit B (CTB) to show that the main inputs to the NAcC and the lateral NAcSh arose from the cortex and olfactory brain regions and to a lesser extent from the thalamus, hippocampus and midbrain. Close to no projections were found from the hypothalamus to the lateral NAcSh and NAcC. In contrast, the main inputs to the medial NAcSh arose from the hippocampus, hypothalamus, amygdala, thalamus and septum, with inputs also arising from pallidal, midbrain and olfactory regions. Notably, less than 1% of neurons brain-wide projected to both the lateral and the medial part of the NAcSh, indicating strong differences between these two regions (Ma et al., 2020). Another study characterized the brain-wide monosynaptic inputs to the NAc by injecting a retrograde rabies virus system into the NAc in D1R- and D2R-Cre transgenic mice and found a similar picture of inputs to the (overall) NAcSh, characterizing them in a cell-type specific fashion. Overall, the NAcSh received its most predominant input from the hippocampus, while the NAcC received its most extensive input from the cortex (Li et al., 2018). Thus, the NAcSh and in particular the medial NAcSh depicts a unique pattern of inputs.

**Figure 1:**
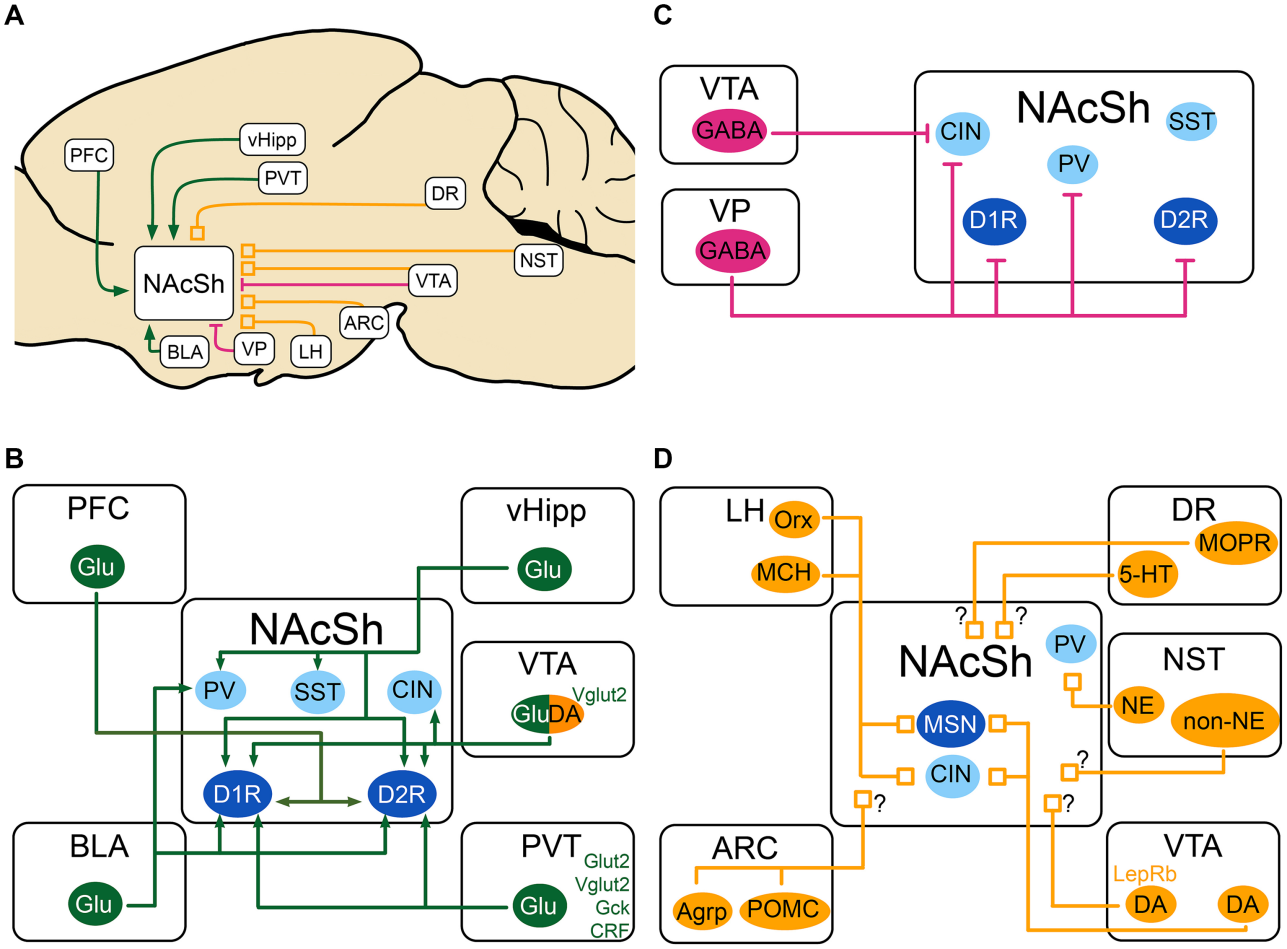
Schematic of the excitatory, inhibitory and neuromodulatory inputs to the NAcSh **(A)** The NAcSh receives excitatory afferents (in green) mainly from the PFC, BLA, PVT and vHipp. **(B)** The VTA and VP are the main source of inhibition (in red) to the NAcSh, while the VTA GABAergic cells mainly inhibit CIN interneurons. **(C)** The local circuitry within the NAcSh is heavily modulated by dopaminergic, noradrenergic, serotonergic and feeding-related inputs (neuromodulatory inputs: in orange). **(D)** The LH sends inputs from Orx and MCH neurons to both MSNs and CIN interneurons, dopaminergic inputs from VTA send denser inputs to CINs compared to MSNs. The identity of the postsynaptic cell of noradrenergic and non-noradrenergic fibers from NST is not known, as for the serotonergic input from DR. There is also an input from the DR that expresses presynaptic MOPR and is modulated by local NAcSh opioids. Agrp, agouti-related protein; ARC, Arcuate nucleus of the hypothalamus; BLA, basolateral nucleus of the amygdala; CIN, cholinergic interneuron; CRF, Corticotropin releasing factor; DA, dopamine neurons; DR, dorsal Raphe nucleus; D1R/D2R, dopamine receptor type 1/2; GABA, GABAergic cells; Gck, glucokinase; Glu, glutamatergic cells; Glut2, Glucose transporter 2; LepRb, leptin receptor; LH, lateral hypothalamus; MCH, melanin- concentrating hormone; MOPR, mu-opioid peptide receptor; MSN, medium spiny neuron; NAcSh, nucleus accumbens shell; NE, norepinephrine; NST, nucleus of the solitary tract; Orx, orexin; PV, parvalbumin fast-spiking neurons; PFC, prefrontal cortex; POMC, pro-opiomelanocortin; PVT, paraventricular nucleus of the thalamus; SST, somatostatin positive interneurons; Vglut2, vesicular glutamate transporter; vHipp, ventral Hippocampus; VP, ventral pallidum; VTA, ventral tegmental area; 5-HT, serotonin. MSNs are shown in dark blue. Interneurons are shown in light blue. When the target cell is unknown, the input is shown with a “?”.

In the next sections, we present the inputs to the NAcSh that are most relevant to feeding behavior. As depicted in Figure 1, the NAcSh integrates feeding-related excitatory inputs from the ventral hippocampus (vHipp), basolateral amygdala (BLA), paraventricular nucleus of the thalamus (PVT) and medial prefrontal cortex (PFC), as well as inhibitory inputs from the ventral tegmental area (VTA) and ventral pallidum (VP). The activity of the NAcSh is further modulated by molecules and neuropeptides from the VTA, lateral hypothalamus (LH), arcuate nucleus of the hypothalamus (ARC), dorsal raphe (DR), and nucleus of the solitary tract (NST).

### 2.1 Excitatory inputs

#### 2.1.1 Excitatory inputs from the ventral hippocampus

One key excitatory input to the NAcSh originates in the vHipp, an important brain region for memory, associative fear learning but also goal-directed behavior and reward seeking. The vHipp sends dense projections to the medial part of the NAcSh and in the context of feeding is generally thought to promote food seeking (O’Donnell and Grace, 1995; Britt et al., 2012; Baimel et al., 2019). The vHipp to NAcSh circuit is known to be sex-specific (Copenhaver and LeGates, 2024). At a neuroanatomical level, optogenetic circuit mapping studies found that vHipp inputs to the NAcSh are by far the largest excitatory input to this region as compared to BLA and PFC excitatory inputs (Do-Monte et al., 2017). Interestingly, in an *in vivo* study, authors found that a majority of NAcSh cells were responsive to vHipp inputs, which served to gate sparser incoming PFC inputs (O’Donnell and Grace, 1995). Others studies used anterograde tracing combined with electron microscopy, finding that NAc neurons receive convergent input from the ventral subiculum of the hippocampus (vSub) and the PFC onto their distal dendrites (French and Totterdell, 2002). Moreover, a similar experiment found that vSub and BLA inputs also converge onto individual dendrites in NAcSh cells (French and Totterdell, 2003). Hence multiple excitatory inputs converge onto the same individual NAcSh cells targeted by vHipp inputs.

Monosynaptic rabies tracing studies also found that vHipp cells target both D1R and D2R-MSNs, finding no significant differences in the preference for either MSN subtype (Scudder et al., 2018). However, optogenetic tracing studies revealed that vHipp inputs generated larger light-evoked excitatory post-synaptic currents (EPSCs) at D1R-MSNs compared to D2R-MSNs (MacAskill et al., 2014; Scudder et al., 2018), suggesting that the synaptic connections of fibers originating in the vHipp are stronger at D1R-MSNs. Moreover, vHipp cells also target PV+ and SST+ interneurons. Indeed, optogenetic activation of vHipp inputs to the medial NAcSh was found to elicit short-latency EPSCs, suggesting a direct excitatory connection from vHipp fibers to MSNs. It also elicited delayed excitatory post-synaptic currents (IPSCs) in D1R-MSNs and D2R-MSNs, suggesting a connection of vHipp inputs to local interneurons which in turn inhibit MSNs, leading to the delayed IPSCs. Furthermore, authors found that the connectivity of the vHipp in the NAcSh was highest with PV+ cells, followed by D1R-MSNs, D2R-MSNs and SST+ interneurons (Scudder et al., 2018).

At the behavioral level, multiple studies showed that vHipp projections to the NAcSh regulate reward- and feeding-related behaviors. Using *in vivo* photometry recordings, Reed et al. found that the activity of vHipp inputs in the NAcSh reduces during feeding. In turn optogenetic inhibition of these inputs strongly promoted feeding, suggesting that vHipp-NAcSh cells normally inhibit food consumption (Reed et al., 2018). Other studies indicated that optogenetic stimulation of the same pathway promotes instrumental responding and real-time place preference (Britt et al., 2012), as well as food palatability, i.e., liking of foods (Yang et al., 2020). Thus, the role of vHipp inputs to the NAcSh is potent but complex and multifaceted, which likely reflects a mix of context- and cellular-subtype-dependent responses.

In sum, the vHipp sends dense projections to the NAcSh and promotes instrumental responding and food palatability but inhibits food consumption. This is the densest excitatory projection within the medial NAcSh, many of which come from the vSub. These inputs converge with PFC and BLA inputs and target PV+ cells as well as D1R- and D2R-MSNs.

#### 2.1.2 Excitatory inputs from the paraventricular nucleus of the thalamus

One other strong excitatory input conveying feeding-related information to the NAcSh originates in the PVT. This midline thalamic nucleus is known to monitor changes in internal states, like glucose homeostasis, arousal or stress, and in turn guides adaptive behaviors like reward seeking via its projections to the NAcSh (Penzo and Gao, 2021). At the neuroanatomical level, studies have shown a complex region-specific architecture, whereby the anterior PVT (aPVT) preferentially targets the dorsomedial NAcSh, while the posterior PVT innervates the ventromedial NAcSh, as well as the bed nucleus of stria terminalis (BNST) and the central amygdala, partly through collaterals (Dong et al., 2017). All cells projecting to the BNST targeted the medial NAcSh as well. Studies also found that inputs coming from the BLA did not synapse onto the same NAcSh neurons that receive PVT input (Lafferty et al., 2020; Zhou et al., 2022). Moreover, studies have shown that orexin and cocaine-and-amphetamine-regulated transcript (CART) fibers from the LH and other hypothalamic nuclei synapsed onto the same neurons in the PVT that project then to the NAcSh (Parsons et al., 2006; Li et al., 2024), indicating that the NAcSh has indirect access to metabolic information via the PVT. Both D1R-MSNs and D2R-MSNs receive glutamatergic inputs from the PVT as shown in one study where 60% of PVT-targeted cells in the NAc were D1R-MSNs while 30% of them were D2R-MSNs (Zhou et al., 2022).

Interestingly, PVT fibers make excitatory synapses onto NAcSh spines where dopaminergic tyrosine hydroxylase positive (TH+) inputs also synapse onto (Pinto et al., 2003). Both inputs bidirectionally influence each other whereby PVT glutamatergic inputs increases DA release without affecting VTA DA firing patterns (Parsons et al., 2007) and conversely DA inputs reduce PVT-mediated excitatory inputs (Christoffel et al., 2021).

At the behavioral level, studies found that the PVT-to-NAcSh projection strongly influences food consumption although the effects are complex. While some studies showed that the PVT-to-NAcSh pauses during feeding and inhibits consumption (Reed et al., 2018; Kessler et al., 2021) or dampens reward seeking during omission of rewards (Do-Monte et al., 2017), other studies found that the PVT-NAcSh projection drives reward consumption or seeking (Labouèbe et al., 2016). This may be due to the heterogeneity of PVT cells projecting to the NAcSh. Indeed, a research group identified a glutamatergic population of neurons in the aPVT that are positive for Glut2, a glucose transporter, and of which over 90% send excitatory projections to the NAcSh. These neurons are activated by low glucose concentrations, send excitatory projections targeting MSNs in the NAcSh and favor food seeking (Labouèbe et al., 2016). The same research group showed that another non-overlapping glutamatergic neuronal population in the aPVT expresses glucokinase (Gck), a glucose sensor and enzyme for glycogenesis, and has somewhat opposite effects on feeding: it inhibits food consumption. Gck + cells were found to send glutamatergic projections to both NAcSh and NAcC. In contrast to aPVT^Glut2^ neurons, aPVT^Gck^ cells were primarily inhibited by low glucose concentrations (Kessler et al., 2021). Strikingly, although these two populations had distinct glucose-sensing mechanisms (inhibited vs. excited) and initiated antagonistic food-seeking behaviors, they both sent glutamatergic projections to the NAcSh. Therefore, it is possible that these inputs converge onto different cell types within the NAcSh region, or alternatively that these inputs are further modulated by an unknown gating mechanism. Another glutamatergic neuronal population in the aPVT that expresses corticotropin-releasing factor (CRF) was shown to send dense monosynaptic projections to the medial NAcSh and control the balance between approach-avoidance behaviors towards food (Engelke et al., 2021). It remains unknown whether there is an overlap between aPVT^CRF^ neurons and aPVT^Glut2^ or aPVT^Gck^ cells, or whether these represent distinct neuronal populations in the PVT.

In sum, aPVT and pPVT send dense projections to the medial NAcSh in a topographical manner, which seem to target both D1R- and D2R-MSNs and interact closely with VTA DA projections at a local level. Part of these projections include glucose sensitive Glut2+ and Gck+, as well as CRF+ PVT neurons, which all regulate feeding behavior but in partially opposite manners.

#### 2.1.3 Excitatory inputs from the basolateral amygdala

Another strong excitatory input to the NAcSh originates in a subnucleus of the amygdala, the BLA, a brain region with key roles in anxiety, valence coding (=firing to conditioned stimuli of positive and negative valence) (Pignatelli and Beyeler, 2019) and reward behaviors. This circuit is known to show sex differences (Truckenbrod et al., 2023). At the neuroanatomical level, Kirouac and Ganguly (1995) showed that fibers coming in from the caudal BLA appear to terminate in the NAcSh in the proximity of MSNs that project to the LH, suggesting a role in feeding. MacAskill and colleagues also used optogenetically-assisted circuit mapping techniques in the medial NAcSh, showing that both D1R- and D2R-MSNs receive BLA input of similar strength (MacAskill et al., 2014). Accordingly, a quantitative analysis of the proportion of BLA inputs to the NAcSh using monosynaptic rabies tracing found no significant difference between inputs to D1R- and D2R-MSNs (Li et al., 2018).

Interestingly, Yu et al. (2017) found that excitatory inputs from the BLA evoked excitatory postsynaptic potentials (EPSPs) in MSNs but also in PV+ interneurons. The input, however, arrived first in PV+ cells, resulting in feedforward inhibition of MSNs. In the NAcSh, PV+ interneurons seemed to receive more excitatory inputs compared to MSNs as shown by higher frequency and amplitude of miniature EPSCs. Thus, PV+ interneurons can constrain the spiking of MSNs in response to excitatory inputs from the BLA or other regions (Yu et al., 2017), and in turn fine-tune how incoming BLA signals regulate NAcSh activity.

At the behavioral level, it is generally thought that the BLA favors reward behaviors and reward seeking as shown by lesion or optogenetic studies (Britt et al., 2012; Yang and Wang, 2017). However, similar to the vHipp, behavioral effects are multifaceted. On the one hand, BLA inputs to the NAcSh were found to pause during feeding and their optogenetic inhibition promoted food consumption (Reed et al., 2018). On the other hand, optogenetic activation of BLA-NAcSh inputs promoted instrumental behaviors and real-time place preference, i.e., were pro-reward (Britt et al., 2012). This latter phenotype was further confirmed by recent work showing that NAc MSNs (primarily the NAcSh) receiving excitatory BLA inputs promote positive reinforcement and food seeking via their projections to VTA DA cells and LH glutamatergic cells (Zhou et al., 2022).

In sum, although MSNs receive direct excitatory BLA inputs, BLA inputs have stronger connections with PV+ interneurons in the NAcSh leading to fast-forward inhibition of MSNs. Overall, the BLA to NAcSh projection promotes reward seeking and positive reinforcement but inhibits consumption, similar to the vHipp.

#### 2.1.4 Excitatory inputs from the prefrontal cortex

Another excitatory input to the NAcSh originates in the PFC, a key cortical region involved in decision-making and goal-directed behaviors. At the neuroanatomical level, it was shown that the PFC sends dense projections to the NAcSh – albeit much sparser as compared to projections to the NAcC – with no clear preference for which MSN type (Britt et al., 2012). This circuit has also been shown to be sex-specific (Johnson et al., 2023). This finding was replicated by others (French and Totterdell, 2002; MacAskill et al., 2014; Li et al., 2018). Like the PVT, PFC inputs to the NAcSh are influenced by incoming DA inputs. Brady and O’Donnell (2004) used *in vivo* intracellular recordings of NAc cells and found that recorded EPSCs generated by PFC stimulation were lower in amplitude when VTA DA neurons were simultaneously stimulated. This phenotype was not diminished by a D1R antagonist, but by a D2R antagonist. Hence DA inputs to the NAcSh modulate incoming PFC signals via presynaptic D2Rs. At the behavioral level, PFC inputs to the NAcSh promote reward seeking, similarly to BLA and vHipp inputs. Indeed, Britt et al. showed that optostimulation of PFC, BLA and vHipp to the NAcSh induced real-time place preference, promoting a pro-reward response (Britt et al., 2012).

In sum, the PFC send projections to the NAcSh and promote reward seeking, though projections are less dense than to the NAcC.

Overall, the multiple excitatory inputs to the NAcSh (vHipp, PVT, BLA, PFC) seem to generally converge in how they influence food seeking, reinforcement behavior and food consumption although there are nuances. Although PFC, BLA and vHipp inputs to the NAcSh are generally pro-reward, PVT inputs are generally anti-reward. There are few exceptions to this, which depend on either the molecular makeup of each projection, or the cellular targets and their output projections.

### 2.2 Inhibitory inputs

#### 2.2.1 Inhibitory inputs from the ventral pallidum

The main source of inhibition to the NAcSh comes from the VP, a brain region involved in reward and value processing as well as reward consumption. At the neuroanatomical level, the VP is a structure containing mainly GABAergic cells and a few glutamatergic cells. Recent work has shown that a subpopulation of GABAergic VP cells, ventral arkypallidal (vArky) neurons, sends dense inhibitory projections to the NAcSh (Vachez et al., 2021). This subpopulation is referred to as vArky neurons, as they resemble the dense, net-like morphology of arkypallidal neurons in the globus pallidus that send projections to the DS (Mallet et al., 2012). Monosynaptic rabies tracing showed that about 84% of all D1R-MSNs received this inhibitory input, 80% of D2R-MSNs and only around 30% of CIN and PV+ interneurons (Vachez et al., 2021). At the behavioral level, the VP inhibitory input to the NAcSh was found to increase its activity before and during food consumption and neuronal activity was even found to correlate with duration of consumption. Moreover, activation of this input inhibited NAcSh neurons (Vachez et al., 2021), leading them to ‘pause’ – a necessary event for food consumption (Krause et al., 2010). Excitation of VP-to-NAcSh projections strongly promoted food consumption indicating they represent one of the dominant sources of inhibition necessary for D1R-MSNs to pause to authorize feeding (O’Connor et al., 2015; Vachez et al., 2021).

In sum, the VP sends a strong source of inhibition to the NAcSh via vArky cells, which target most MSNs as well as CIN and PV+ interneurons and promotes feeding via inhibition of NAcSh cells.

#### 2.2.2 Inhibitory inputs from the ventral tegmental area

Another source of inhibition to the NAcSh comes from the VTA, a midbrain region playing critical roles in reward-related behavioral regulation. At the neuroanatomical level, GABAergic cells are interspersed with TH^+^ dopaminergic cells in the VTA (Olson and Nestler, 2007). GABAergic neurons are split between GABA interneurons, that provide local inhibition, and GABA neurons that send long-range projections, including to the NAcSh (Van Bockstaele and Pickel, 1995; Creed et al., 2014). Overall, VTA inputs to the dorsomedial NAcSh comprise about 66% of dopamine TH+ neurons, 25% of GABAergic projection neurons and the rest is glutamatergic (Margolis et al., 2006; Chuhma et al., 2014). VTA GABAergic projections seem to preferentially contact CIN neurons (Brown et al., 2012). At a region-specific level, although one study found that VTA GABA neurons preferentially project to the lateral NAcSh (Beier et al., 2019), another study showed that VTA GABA neurons preferentially project to the ventromedial NAcSh and less to the dorsomedial NAcSh region (Al-Hasani et al., 2021); hence future work is needed to map these projections. At the behavioral level, long-range VTA GABA projections to the NAc were shown to pause CIN firing, reduce cholinergic tone in the NAc, and in turn modulate associative learning (Brown et al., 2012), as well as contribute to reward consumption (van Zessen et al., 2012).

In sum, the VTA sends long-range GABAergic projections to the NAcSh, primarily targeting CINs and modulating associative learning, as well as reward consumption. These projections seem topographically organized, possibly preferring the lateral NAcSh and ventromedial NAcSh as compared to the dorsomedial NAcSh.

### 2.3 Neuromodulatory inputs

#### 2.3.1 Dopaminergic inputs from the ventral tegmental area

The VTA sends very dense dopaminergic projections to the NAcSh (Morales and Margolis, 2017), and plays key roles in associative learning, motivation and effort (Berke, 2018). At a neuroanatomical level, studies have shown a dorso-ventral gradient of release: more dorsal regions of the striatum exhibit higher levels of stimulated DA release, while more ventral ones like the NAcSh show lower levels (Calipari et al., 2012). Of note, DA inputs to the NAcSh are known to be sex-specific and regulated by sex hormone-dependent signaling (Yoest et al., 2019; Brundage et al., 2022). Dopamine acts via D1 to D5 receptors expressed primarily on D1R- and D2R-MSNs, as well as CIN which express D2Rs. Dopaminergic fibers to the medial NAcSh originate in the medial posterior VTA, while projections targeting the lateral NAcSh mainly originate in the lateral VTA and in the Substantia nigra pars compacta (SNc) (Beier et al., 2015). Interestingly, axon collateralization analysis of dopaminergic fibers revealed that axons from the lateral VTA that target the lateral NAcSh send axon collaterals to the NAcC and DS but not to the medial NAcSh. On the other hand, dopamine neurons projecting to the medial NAcSh were found to collateralize in the VP and other outer-striatal territories (Beier et al., 2015). At the behavioral level, although VTA DA neurons do not directly regulate food consumption, they play important roles in learning to associate cues and contexts with food rewards and to code reward expectations, thus promote the establishment of food associations. Moreover, VTA DA neurons regulate the motivation for action and effort, also known as “the value of work,” which impacts the motivation for food seeking and consumption (Berke, 2018).

Importantly, studies have shown that 30% of dopamine neurons in the VTA co-release glutamate within the NAcSh (Stuber et al., 2010; Li et al., 2013; Zhang et al., 2015; Qi et al., 2016; Eskenazi et al., 2021). Dopamine-glutamate co-expressing cells send their projections primarily from the ventromedial VTA mainly to the medial NAcSh as well as to the OT, of which a subset are Aldh1a1+ (Stuber et al., 2010; Li et al., 2013; Zhang et al., 2015; Eskenazi et al., 2021). At the behavioral level, glutamate release from these neurons has been shown to be reinforcing, while dopamine release induced avoidance. Moreover dopamine-glutamate neurons are known to contribute to the attribution of salience to cues or contexts, and thus indirectly contribute to the regulation of food-related associative learning (Mingote et al., 2019; Warlow et al., 2024).

In sum, the VTA sends dense dopamine projections to the NAcSh, acting onto MSNs and CINs to regulate associative learning and motivation. 30% of VTA DA cells projecting to the NAcSh co-release glutamate and regulate salience attribution.

#### 2.3.2 Neuropeptidergic inputs from the hypothalamus

The hypothalamus, the metabolic center of the brain, exerts its influence on the medial NAcSh by directly sending neuropeptidergic projections to the NAc informing primarily on metabolic bodily states and in turn driving homeostatic feeding. The strongest projections from the hypothalamus comes from the lateral hypothalamus (LH), which exerts its influence directly onto the NAcSh via projections. Monosynaptic rabies tracing showed that LH fibers projecting to the NAcSh disproportionally target D1R-MSNs as compared to D2R-MSNs (Li et al., 2018). Since D1R-MSNs project back to the LH, this suggests the existence of a bidirectional communication between NAc D1R-MSNs and the LH. The LH influences the NAcSh via release of melanin- concentrating hormone (MCH) and orexin, two neuropeptides with crucial roles in feeding behavior (Baldo et al., 2003; Diniz and Bittencourt, 2017; Castro and Bruchas, 2019). With respect to MCH, the highest density of MCH+ positive fibers in the NAc were documented in the NAcSh, specifically in the ventromedial and dorsomedial parts, while the NAcC had the lowest density of fibers. Retrograde tracing of MCH fibers with Fluoro-Gold showed that fibers in the NAcSh originate from various hypothalamic nuclei with the highest number found in the LH and perifornical area. Immunohistochemical labeling showed that MCH+ fibers send many collaterals within the NAcSh and make contacts with both MSNs and CIN neurons (Haemmerle et al., 2015). MCH inputs were found to inhibit MSN firing by regulating their excitability via its Gi/o coupled G-protein-coupled receptor MCHR1 (Sears et al., 2010). Furthermore, *in situ* hybridization experiments showed that MCHR1 colocalizes with dynorphin+ cells (primarily D1R-MSNs) and enkephalin+ cells (primarily D2R-MSNs), confirming that both MSN subtypes respond to MCH input (Georgescu, 2005). At the behavioral level, MCH neurons are activated by physiological concentrations of glucose; in turn MCH increases food intake and reduces energy expenditure suggesting a role in energy conservation and accumulation (Concetti et al., 2023). For instance, Terrill et al. showed that activation of MCH receptors in the NAcSh promotes consumption of chow and palatable sucrose in a sex-dependent manner (Terrill et al., 2020). The specific connectivity of MCH with NAcSh projections has not been demonstrated though. Of note, MCH neurons are not specialized in feeding, as they can also affect sleep states, cognition or anxiety (Concetti et al., 2023).

LH orexin neurons also send dense projections to the NAcSh and target mainly D2R-MSNs (Blomeley et al., 2018). Activation of orexin fibers leads to excitation of target cells, which are primarily D2R-MSNs and NPY+ interneurons rather than D1R-MSNs (Burdakov, 2020), partly in contradiction with cell-unspecific tracing studies (Li et al., 2018) (see previous paragraph). Hence the preference for D1R-MSNs vs. D2R-MSNs seems to depend on the LH cellular subtype. Within the LH, all orexin neurons co-express dynorphin (Chou et al., 2001) and some express vesicular glutamate transporter 2 (Vglut2) (Rosin et al., 2003). Of note, these neurons are distinct from LH GABAergic cells. Indeed, vesicular GABA transporter (*Vgat*) positive LH neurons do not co-express orexin nor MCH and importantly, LH GABA neurons do not project to the NAcSh (Sharpe et al., 2017). Interestingly, monosynaptic projections from LH orexin neurons were found to increase the levels of dopamine in the NAcSh region directly (Baldo et al., 2004; Patyal et al., 2012). Moreover, LH orexin neurons also increase the levels of dopamine in the NAcSh indirectly: orexin fibers excite VTA dopamine neurons and two-thirds of the innervated dopamine neurons project to the NAcSh (Kalló et al., 2022). At the behavioral level, orexin neurons are activated by fasting and inactivated during eating or inhibited by specific nutrients such as D-glucose (Peleg-Raibstein et al., 2023). Moreover, inactivation of orexin neurons promotes overeating and obesity, suggesting that orexin cells facilitate energy expenditure and disfavor eating (Hara et al., 2001; González et al., 2016). However, like MCH neurons, orexin neurons are multifaceted and do not only modulate feeding, but also sleep states, arousal or locomotion (Peleg-Raibstein et al., 2023). Whether these various behaviors rely on orexin NAcSh projections or projections to other brain regions remains to be determined.

The arcuate nucleus of the hypothalamus (ARC) also sends homeostatic and feeding- related inputs to the NAcSh. This includes relatively weak inputs from pro-satiety pro-opiomelanocortin (POMC) + neurons which co-release α-melanocyte-stimulating hormone (MSH), cocaine- and amphetamine-regulated transcript (CART) and beta-endorphin as well as from pro-feeding agouti-related protein (AgRP) + neurons which co-release AgRP, neuropeptide Y (NPY) and GABA. Melanocortin-4 receptors (MC4Rs), receptors for MSH, are highly expressed within the NAcSh region, compared to the VTA, where mainly melanocortin-3 receptors (MC3Rs) are expressed (Brog et al., 1993; Dunigan and Roseberry, 2022). At the behavioral level, AgRP neurons are the canonical ‘first-order’ pro-feeding neurons, while POMC are the canonical ‘first-order’ stop-eating neurons, both of which thus strongly encode information about metabolic states like hunger or satiety. More details on POMC and AgRP inputs onto the NAc and other regions can be found in other excellent papers (Pandit et al., 2013; Castro and Bruchas, 2019; Dunigan and Roseberry, 2022).

In sum, the NAcSh receives broad metabolism-related neuropeptidergic inputs from the lateral hypothalamus, as well from the ARC, which includes dense MCH+ and orexin+ input as well as weaker inputs from AgRP and POMC cells.

#### 2.3.3 Serotonergic inputs from the dorsal raphe

The NAc receives extensive serotonergic (5-HT) innervation from the DR as shown by Parent et al. using a autoradiography technique (Parent et al., 1981; Brog et al., 1993). 5-HT was shown to have modulatory effects on glutamatergic inputs to the NAcSh. Optogenetic stimulation of individual input fibers from the PFC, PVT, BLA and vHipp are known to generate EPSCs in NAcSh MSNs. Application of 5-HT largely reduced PVT-, BLA- and vHipp-driven EPSCs in NAcSh MSNs, but had no effect on PFC-driven EPSCs, indicating a possible neuromodulatory role of serotonin in dampening excitatory inputs to the NAcSh; its precise role remains unclear though (Christoffel et al., 2021). At the behavioral level, the 5-HT system has long been known to contribute to both homeostatic feeding and hedonic feeding. From a homeostatic perspective, 5-HT promotes the inhibition of food intake in the fed state, whereas from a hedonic perspective, reports are conflicted with some studies reporting suppressed motivation-based food consumption and some reporting an increase; this depends on which serotonin receptor subtype is activated within the NAcSh (Pratt et al., 2009; van Galen et al., 2021; Anderson et al., 2023).

In sum, dense 5-HT inputs to the NAcSh from the DR modulate incoming excitatory inputs within the region.

#### 2.3.4 Opioid-related inputs

The NAcSh also receives rich innervation from the opioid system including projections arising from the DR or VP (Urstadt et al., 2013; Castro et al., 2021). Opioids can act through four receptors: mu opioid receptors (MOPRs), delta opioid receptors (DOPRs), kappa opioid receptors (KOPRs) and nociceptin receptors (NOPRs), all of which are present in the NAcSh (for an excellent review see Castro and Bruchas, 2019). In the context of eating and reward behavior, KOPR and MOPR play particularly important roles. MOPRs are perhaps the most well-described in the context of appetitive motivation with many MOPR agonists (e.g., morphine) known to induce potent pro-reward effects. MOPR stimulation in the NAc favors motivated behaviors including increased feeding or operant responding to food rewards but also drug-seeking or conditioned place preference (Bakshi and Kelley, 1993; Peciña and Berridge, 2000; see also Castro and Bruchas, 2019). Research investigating the mechanisms of actions of opioids in the NAcSh showed that the DR provides a key contribution (Castro et al., 2021). Authors showed that locally released enkephalin in the NAcSh in hungry mice acts on presynaptic MOPR on DR projections to the NAcSh which in turn promotes eating. Moreover, independent of its effects on food intake, MOPR also modulates hedonic responses, and does so in a topographical manner: MOPR stimulation in the rostrodorsal NAcSh promotes “liking,” while in the caudal NAcSh it suppresses it (Pecina, 2005). In addition, KOPR, although primarily known in the context of aversion behaviors and stress, has been increasingly implicated in appetitive motivation as well. Early studies demonstrated that loss of KOPR function within the NAcSh prevents food-deprived stimulated intake (Bodnar et al., 1995). More recent experiments using selective KOPR agonists highlighted anatomical differences, where stimulation in the rostral NAcSh increased positive hedonic reactions, while stimulation in the caudal NAcSh reduced them, akin to the effects of MOPR (Castro and Berridge, 2014b). Moreover, optogenetic studies showed that the KOPR system can directly generate place preference or avoidance: indeed, stimulation of dynorphin+ neurons, which release dynorphin acting on KOPRs, generated either place preference or avoidance, depending on the stimulated site along the dorso-ventral axis (Al-Hasani et al., 2015, 2018).

In sum, MOPR and KOPR play key roles in the NAcSh in the context of feeding: MOPR promotes food consumption, while both MOPR and KOPR modulate appetitive hedonic-like responses (‘liking’): they promote them in the rostral NAcSh and inhibit them in the caudal NAcSh.

#### 2.3.5 Noradrenergic inputs from the nucleus of the solitary tract

Another neuromodulatory afferent to the NAcSh is the catecholamine input from the norepinephrine (NE) cell population arising from the NST, a brain region with key roles in feeding behavior (Grill and Hayes, 2012). Sparse projections also arise from the NE groups in the locus coeruleus (LC) and the region of the caudal ventrolateral medulla (CVLM). NE is synthesized mainly in dopamine- β- hydroxylase (DBH)- containing cells and immunohistochemical experiments in brain slices show few DBH+ fibers in the DS and NAcC. On the other hand, the NAcSh receives overall dense NE-innervation (Berridge et al., 1997; Delfs et al., 1998). Delfs et al. (1998) showed that rostral LC and NST send both TH+ and TH- cells to the NAcSh, as shown by retrograde tracing. This indicated that these structures send both NE and non-NE projections to the NAcSh. However, in later studies, authors found that only few NE cells in the LC projected to the NAcSh. Rather, NE projections to the NAcSh originated primarily from the NST instead of the LC, and primarily targeted the caudal part of the NAcSh, as shown by anterograde tracing studies (Delfs et al., 1998). Recent work also found that NE influences the activity of local interneuron populations within the NAcSh, in particular PV+ interneurons. Authors used whole-cell patch clamp recordings showing that NE fibers in the NAcSh promote feed forward inhibition from PFC inputs to MSNs by inhibiting PFC glutamatergic transmission onto PV interneurons (Manz et al., 2021). At the behavioral level, it remains unclear how the specific NE projections from the NST to the NAc regulate food consumption and reward behavior and thus this remains to be studied in detail.

In sum, the NST and other NE regions project to the NAcSh via NE and non-NE projections; the NE projection to the NAcSh primarily arises from the NST and is most dense in the caudal part of the NAcSh; although its definite role in feeding and reward behaviors remains to be addressed.

### 2.4 Relevant metabolic receptors within the accumbens shell

In addition to projections from the hypothalamus that convey metabolic and homeostasis relevant information, the NAcSh also expresses a number of metabolic receptors that are capable of sensing the levels of relevant peripheral hormones such as insulin, ghrelin and to some extent GLP-1. Insulin, a pancreatic hormone which regulates glucose blood levels, can pass the blood–brain barrier (BBB) via trans-endothelial transport, and there is a particularly high number of insulin receptors in the NAc. Studies have shown that insulin can facilitate evoked DA release via CINs, or excite MSNs, and behaviorally it modulates reward value (Stouffer et al., 2015; Fetterly et al., 2021; Carr and Weiner, 2022). The hunger-related hormone ghrelin can cross the BBB and there are receptors for ghrelin in the NAcSh (albeit much less than in the VTA). Moreover, ghrelin infusion into the NAcSh promotes food intake and motivation, although some of these effects may occur indirectly via the VTA (Skibicka et al., 2011; Cone et al., 2015; Vestlund et al., 2019). Finally, although there are functional receptors for the satiety-related gut hormone GLP-1 in the NAc (Chen X. Y. et al., 2021), and GLP-1R agonists can modulate NAc activity (Labouesse et al., 2012), it is unlikely that naturally released gut GLP-1 can pass the BBB and reach the NAc (Buller and Blouet, 2024); rather NAc GLP-1R are likely targeted instead by GLP-1-expressing neurons from the NST (Chen X. Y. et al., 2021).

Moreover, the adipokine leptin, which primarily acts in the LH and ARC to decrease food intake, has little to no receptors to bind at within the NAcSh (Leshan et al., 2010). However this hormone can influence the NAcSh indirectly via the VTA by modulating the activity of VTA neurons and ultimately DA release in the NAcSh. In leptin-deficient obese mice, the absence of leptin resulted in reduced TH levels and decreased evoked DA release in the NAcSh, leading to reduced dopamine-dependent behaviors such as amphetamine-evoked locomotion. Whether this phenotype also drives altered reward seeking or eating remains to be determined (Fulton et al., 2006).

In sum, insulin, ghrelin, to some extent GLP-1, and indirectly leptin have the potential to modulate the NAcSh to bring metabolism related information and in turn affect feeding behavior.

## 3 Outputs of the nucleus accumbens shell

**Figure 2:**
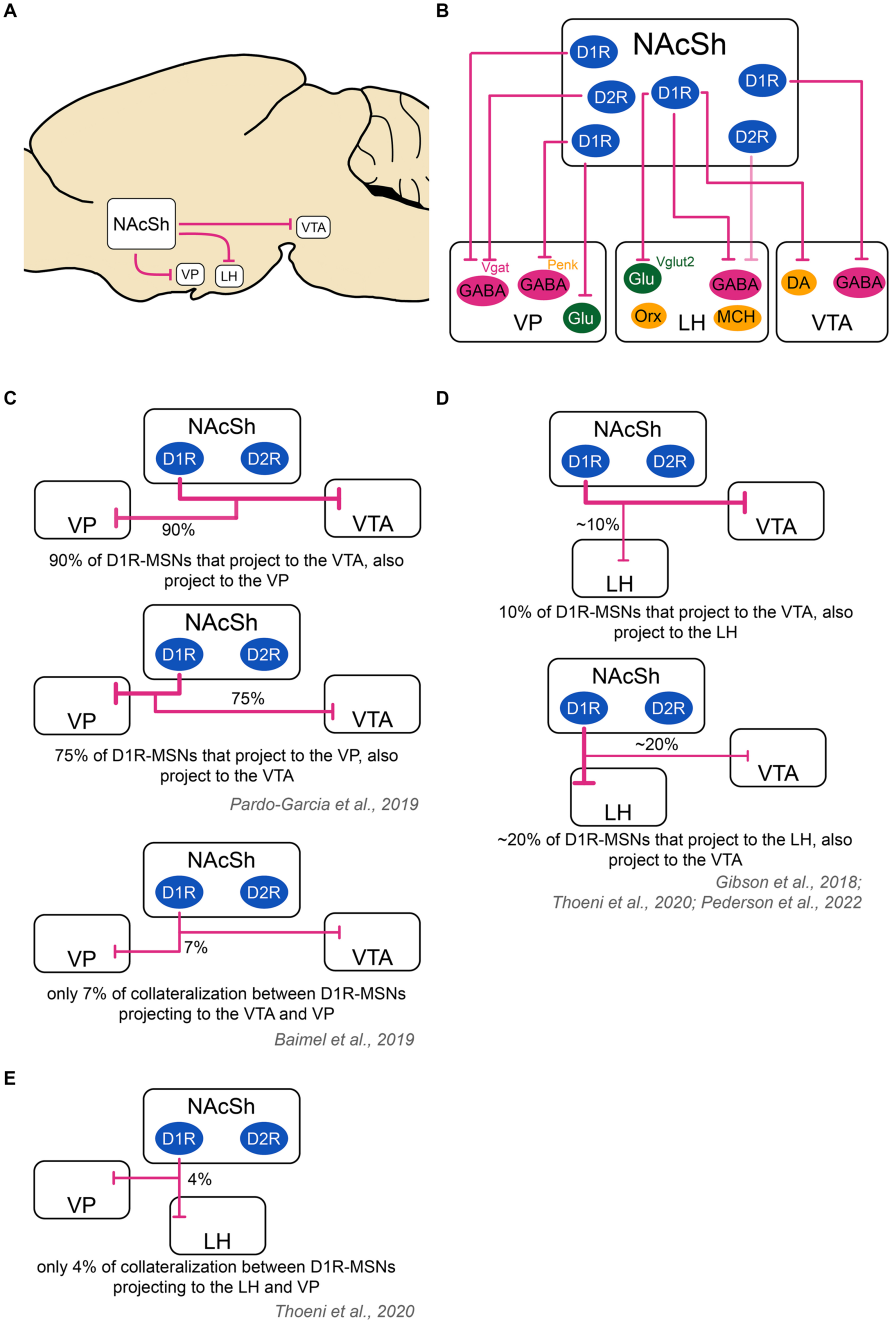
Schematic of the outputs of the nucleus accumbens shell to downstream structures and collateralization patterns. **(A)** The main output targets of the NAcSh are the VP, LH and VTA. **(B)** D2R-MSNs send projections mainly to GABAergic neurons in the VP. They also send very weak projections to the LH [target cell unknown as shown with a ‘?’; see (O’Connor et al., 2015)] and likely even weaker projections to the VTA [target cell unknown as shown with a ‘?’; see (Bocklisch et al., 2013; Matsui et al., 2014; Kupchik et al., 2015; Edwards et al., 2017; Gibson et al., 2018; Yang et al., 2018; Bond et al., 2020)]. D1R-MSNs project to the VP, LH and VTA. In the VP, D1R-MSNs target GABAergic neurons, including those that are Vgat+ and those that are Penk+, as well as glutamatergic neurons. In the LH, D1R-MSNs target GABAergic Vgat+ neurons and glutamatergic vglut2+ neurons, but not orexin or MCH neurons. In the VTA, D1R-MSNs target both DA and GABA neurons. Color code: green: excitatory cells, pink: inhibitory cells, yellow: neuromodulatory cells. **(C)** D1R-MSNs projecting to the VP and VTA might strongly overlap. In one study, it was found that 94% of D1R-MSNs projecting to the VTA also projected to the VP, while 70% of D1R-MSNs projecting to the VP also projected to the VTA (Pardo-Garcia et al., 2019). In another study using a different technique, there was only a 7% overlap between VP and VTA D1R-MSNs projections (Baimel et al., 2019). These contradictory results warrant further study. **(D)** D1R-MSNs projecting to the LH and VTA show a minor overlap. An estimate ~10% of cells projecting to the VTA also projected to the LH. An estimate ~20% of cells projecting to the LH also projected to the VTA (Gibson et al., 2018; Thoeni et al., 2020; Pedersen et al., 2022). **(E)** D1R-MSNs projecting to the VP and LH show poor overlap: on average, 4% of cells projecting to the VP and LH co-localized (Thoeni et al., 2020). The thickness of the arrows represents the relative amount of input. DA, dopamine neurons; D1R/D2R, dopamine receptor type 1/2; GABA, GABAergic cells; Glu, glutamatergic cells; LH, lateral hypothalamus; MCH, melanin- concentrating hormone; NAcSh, nucleus accumbens shell; Orx, orexin; Penk, Proenkephalin; Vgat, vesicular GABA transporter; Vglut2, vesicular glutamate transporter; VP, ventral pallidum; VTA, ventral tegmental area.

Now that we provided an overview of the inputs to the NAcSh, we summarize recent findings regarding the connectivity of the NAcSh to its downstream target regions, primarily the VTA, LH and VP, as shown in Figure 2. After describing these connections, we discuss their functional role in feeding behavior.

### 3.1 Output projections to the ventral tegmental area

The NAcSh exhibits differential projections to various brain regions, including the VTA to which primarily D1R-MSNs project to (O’Connor et al., 2015; Gibson et al., 2018). Although some studies did describe D2R-MSNs projections to the VTA, levels are always very low (Bocklisch et al., 2013; Matsui et al., 2014; Kupchik et al., 2015; Edwards et al., 2017; Gibson et al., 2018; Yang et al., 2018; Bond et al., 2020), indicating that if the D2R-MSN projection exists, they are minor. D1R-MSNs project to both VTA DA and VTA GABA neurons as shown with retrograde tracing studies (Beier et al., 2015) and electrophysiology (Yang et al., 2018). For instance, optogenetic circuit mapping work found small IPSCs detected in two-thirds of all VTA dopamine neurons after D1R-MSN stimulation, while large IPSCs were recorded in all VTA GABAergic cells; hence D1R-MSNs preferentially inhibit VTA GABAergic neurons (Xia et al., 2011; Bocklisch et al., 2013). In another study looking at the entire NAc, GABA_B_R antagonists blocked D1R-MSN-evoked IPSCs in VTA DA neurons, while GABA_A_R antagonists blocked D1R-MSN-evoked IPSCs in VTA GABA neurons (Edwards et al., 2017), indicating that both GABA_A_R and GABA_B_R contribute to the effects of D1R-MSNs in the VTA.

Connections between the lateral and medial NAcSh and the VTA show an anatomical segregation and the existence of closed loops between the two brain regions. Yang et al. (2018) found that medial NAcSh D1R-MSNs targeted DA neurons that project back to the medial NAcSh, while lateral NAcSh D1R-MSNs targeted GABA neurons in the VTA which in turn inhibit DA neurons that project back to the lateral NAcSh. Furthermore, MSNs in the medial NAcSh compared to lateral NAcSh exerted direct inhibitory control over two distinct subpopulations of mesolimbic DA neurons by activating different subtypes of GABA receptors. Specifically, D1R-MSNs in the medial NAcSh preferentially suppressed medial NAcSh-projecting DA neurons through GABA_A_R and lateral NAcSh-projecting DA neurons through GABA_B_R. Moreover, D1R-MSNs in the lateral NAcSh primarily targeted GABAergic interneurons in the VTA, rather than DA neurons, leading to an overall disinhibition of lateral NAcSh-projecting DA neurons (Edwards et al., 2017; Yang et al., 2018). Hence, different subsections of the NAcSh affect the activity of VTA DA vs. GABA neurons differentially.

D1R-MSNs projecting to the VTA also heavily collateralize in the VP: In one study, more than 90% of D1R-MSNs projecting to the VTA sent collaterals to the VP while three-quarters of D1R-MSNs projecting to the VP sent collaterals to the VTA (Pardo-Garcia et al., 2019). This finding was contradicted by another study finding only 7% overlap between VP- and VTA-projecting D1R-MSN populations (Baimel et al., 2019), warranting further work. On the other hand, D1R-MSNs projecting to the VTA only sparsely collateralize in the LH: in one study, authors found that VTA and LH projections originated from distinct cells in the NAcSh, with less than 20% of D1R-MSNs projecting to both regions via collaterals (Gibson et al., 2018).

In sum, the NAcSh sends dense D1R-MSNs projections to the VTA, targeting both DA and GABA neuron populations. D1R-MSN projections from the medial NAcSh and lateral NAcSh are topographically organized within the VTA, where NAcSh cells target DA neurons that back-project to the same input NAcSh input region. NAcSh projections to the VTA overlap to a possibly large extent with NAcSh VP projections, but overall, relatively weakly with LH projections. The behavioral significance of NAcSh to VTA projections are discussed in a later section.

### 3.2 Output projections to the lateral hypothalamus

The LH is a large subnucleus within the hypothalamus that receives input from many structures, including the NAc. Projections to the LH arising in the NAc originate mainly in the NAcSh part (Heimer et al., 1991). Indeed, anterograde tracers injected at different coordinates in the NAcC and in the NAcSh have shown that the medial section of the NAcSh projects to the LH, whereas the ventrolateral NAcSh and NAcC do not, or only sparsely (Usuda et al., 1998). The LH encompasses a notable diversity of cell types: it contains inhibitory GABA neurons, glutamatergic neurons, excitatory orexin neurons that co-express dynorphin and vesicular glutamate transporters (Chou et al., 2001; Rosin et al., 2003), and MCH neurons. Which cells are targeted by the NAcSh? Using cFos expression, a marker for neuronal activity, it was shown that infusion of a GABA agonist into the NAcSh leads to Fos expression in orexin and MCH neurons within the LH (Baldo et al., 2004) and optogenetic stimulation of NAcSh neurons and NAcSh fibers in the LH reduced cFos expression by 50% in the LH, primarily in LH orexin neurons (Larson et al., 2015). This study indicated that orexin neurons are either directly inhibited by the NAcSh or indirectly via polysynaptic circuits. Later, optogenetic and circuit-based mapping tools were used to describe the input of MSNs to the LH: this revealed that none of the 29 cells that responded with IPSCs after NAcSh optogenetic stimulation were positive for orexin or MCH, suggesting they do not receive direct input (O’Connor et al., 2015). Instead it was found that NAcSh cells target both GABA neurons and glutamatergic neurons in the LH (O’Connor et al., 2015; Thoeni et al., 2020), a finding later confirmed by others (Zhou et al., 2022). Moreover, this projection involves at least in part GABA_A_ receptors in the LH as the effects of NAcSh inhibition on feeding is prevented by concomitant infusion of GABA_A_ receptor agonist into the LH (Maldonado-Irizarry et al., 1995; Urstadt et al., 2013). Interestingly, it was shown that of all NAcSh projection neurons monosynaptically connected with LH Vgat neurons, near 100% of them were D1R-MSNs rather than D2R-MSNs (O’Connor et al., 2015; Thoeni et al., 2020). Only a very small proportion of D2R-MSNs projected to the LH, mainly arising from the caudal NAcSh (O’Connor et al., 2015; Thoeni et al., 2020).

MSNs projecting to the LH (mostly D1R-MSNs) only have sparse collaterals with the VP: indeed, a study found that only 4% of D1R-MSNs sent collaterals to the LH and VP (Thoeni et al., 2020). Furthermore, 10% of D1R-MSNs sent projections to both LH and VTA validating the only minor overlap between these NAcSh MSN populations (Thoeni et al., 2020), which was also confirmed by others (Gibson et al., 2018; Pedersen et al., 2022).

In sum, the NAcSh projects to the LH, primarily via D1R-MSNs which target LH GABA and LH glutamatergic cells, rather than orexin and MCH cells. LH-projecting D1R-MSNs overlap weakly with VTA and VP projecting cells. The behavioral significance of NAcSh to LH projections are discussed in a later section.

### 3.3 Output projections to the ventral pallidum

The VP is a ventral brain region belonging to the limbic basal ganglia, that receives input from both the core and the shell regions of the NAc (Gendelis et al., 2021). The NAcSh preferentially projects to the ventromedial VP, while the NAcC sends afferents mainly to dorsolateral regions of the VP, following a ventromedial to dorsolateral topology (Haber et al., 1985; Heimer et al., 1991). Both D1R-and D2R-MSNs from the NAcSh send efferents to the ventromedial VP and connect with overlapping populations as shown by Creed et al. (2016). In this study, CTB injection into the ventromedial part of the VP in D1R-tdTomato or D2R-tdTomato mice showed that more than 90% of ventromedial VP cells were targeted by D1R-MSNs, while 75% received input from D2R-MSNs. IPSCs were mediated through GABA_A_R (Creed et al., 2016; see also Kupchik et al., 2015). The VP is primarily composed of Vgat+ GABA cells (74%, subset of which are either PV+ or Penk+), glutamatergic cells (15%) and CIN (11%) (Faget et al., 2018). Cell-type specific connectivity experiments showed that D1R-MSNs preferentially innervate VP glutamatergic cells and VP GABAergic Penk+ cells, whereas VP GABA that are Penk- and VP PV+ neurons received approximately the same level of innervation from D1R- and D2R-MSNs (Knowland et al., 2017; Heinsbroek et al., 2020).

In sum, the NAcSh projects to the ventromedial VP via equal proportions of D1R-MSNs and D2R-MSNs, which in turn target partly non-overlapping cells in the VP. As mentioned above, VP-projecting NAcSh cells may overlap with VTA-projecting cells but they overlap very weakly with LH-projecting cells. The behavioral significance of NAcSh to VP projections are discussed in a later section.

Finally, it is worth mentioning that NAcSh projections not only release the fast neurotransmitter GABA at their presynaptic terminals, but also opioids: D1R-MSNs release dynorphin (KOPR ligand) while D2R-MSNs release enkephalin (DOPR ligand) (Creed et al., 2016; Soares-Cunha et al., 2020). For instance, opioid agonism in the VP increases hedonic reactions to sucrose rewards and stimulates food consumption (Castro and Berridge, 2014a; Inui and Shimura, 2014; Creed et al., 2016).

In conclusion, the main output regions of the NAc are the VTA, LH and VP where both GABA and opioid neuropeptides are released. The VTA and LH receive primarily D1R-MSN input, while the VP receives input from both D1R- and D2R-MSNs. While VTA- and LH-projecting NAcSh D1R-MSNs are mostly considered to be separate neural populations (low collateralization), the extent to which VTA- and VP-projecting D1R-MSNs overlap remains to be determined. Of note, studies have also suggested that the anterior NAcSh projects not only to the LH, but also to the lateral preoptic area involved in sleep, thirst and reward and weakly to the lateral septum involved in fear, anxiety, memory and reward (Zahm et al., 2013). The anatomical extent and functional relevance of these projections remain to be determined.

## 4 The role of nucleus accumbens shell projection neurons in food intake

Classically, food intake regulation is thought to occur primarily within the hypothalamus, the metabolic center of the brain and main regulator of homeostatic need-based feeding. However, in the past two decades there has been a resurgence of interest for other brains regions, in particular those regulating hedonic aspects of feeding. The NAcSh is of particular interest in this context as it is embedded within both homeostatic and hedonic feeding circuits, integrating information from both sources to modulate behavioral output. In the previous sections, we reviewed the inputs to the NAcSh and the outputs of NAcSh projection neurons. We now discuss the type of food-related signals that can be detected within NAcSh cells and in turn how the NAcSh contributes to feeding behavior in living animals.

The NAcSh integrates signals purely related to metabolic need (e.g., NST and hypothalamic inputs), sensory, arousal, context, associative learning and reward-related signals (from the vHipp, PVT, BLA, PFC, VTA, VP) and palatability/liking related signals (from the vHipp and opioid systems for example), which we presented in detail above. At the output level, however, the most unequivocal demonstration of the functional role of NAcSh projection neurons seem to primarily involve the control of the onset and offset of food consumption independent from metabolic need [see, e.g., (Stratford and Kelley, 1997; Taha and Fields, 2006; Krause et al., 2010; O’Connor et al., 2015)]; with less studied or less clear roles in other related behaviors. For example a recent study showed that NAcSh projection neurons do not affect food liking (food palatability), even though vHipp inputs to the NAcSh do (Yang et al., 2020). Therefore, in the next section we primarily discuss how NAcSh mediates food consumption. Future studies and reviews are needed to outline the possibly more complex role of NAcSh projection neurons in other food-related behaviors.

The prominent role of the NAcSh in food consumption – in particular the medial part – was established with several eminent pharmacological studies in the 1990s. Studies had shown that inactivating NAc neurons via GABAR agonist infusion into the medial NAcSh, but not the NAcC or lateral NAcSh region, dramatically increased food intake in *ad libitum* fed rats, whereas drinking was not affected (Stratford and Kelley, 1997). Similarly, infusion of a glutamate AMPA receptor antagonist into the medial NAcSh also increased food intake in satiated rats. This effect was abolished by applying muscimol (a GABAR agonist) into the LH, suggesting that the projections to the LH mediate the feeding effects of the medial NAcSh (Maldonado-Irizarry et al., 1995). Interestingly, an anterior–posterior gradient effect was observed: GABAR agonist injection into the anterior and medial NAcSh resulted in increased consumption, whereas infusion into the posterior medial NAcSh decreased food consumption and promoted aversive behaviors like defensive burying behaviors (vigorous treading-like movements of the forepaws that splash sawdust toward a threatening object) (Reynolds and Berridge, 2001). These historical studies (and others) led to a large number of studies investigating (i) how the NAcSh monitors food-related signals *in vivo* and (ii) how the NAcSh regulates food intake behavior.

### 4.1 Food-related behavioral information encoded by NAc shell MSNs

We here describe studies looking at behaviorally evoked activity in NAcSh MSNs. Fields and colleagues conducted a series of *in vivo* single-unit recording studies to determine the electrophysiological activity of the NAc (core+shell) during the consumption of rewarding solutions varying in sucrose concentration from 0 to 10%. Excitatory and inhibitory responses were detected in both the NAcC and NAcSh. About 30% of all units (single neurons recorded) responded with short-lived excitation during food consumption, which occurred with short latency at consumption onset. In some of the units, excitatory responses increased in magnitude with increased reward palatability (percentage of sucrose), while in other units, excitatory responses increased in the presence of preferred rewards (as assessed on a preference test). Responses did not significantly vary with metabolic state (satiety). This suggested that units with excitatory NAc responses monitor both the sensory properties (palatability) and relative value (preference) of rewards (Taha and Fields, 2005). On the other hand, 70% of all units responded with prolonged inhibitions, rather than excitations, during food consumption. The timing of inhibitions was different, with inhibition beginning during the approach phase, shortly before mice started licking, and stopped when mice stopped licking. These inhibitory responses also occurred when animals licked but rewards were not delivered, and were not modulated by the sensory properties or value of the rewards, nor did they correlate with locomotion outside of consumption (Taha and Fields, 2005). Other studies also found that inhibitory responses tightly correlated with the electromyography activity of the mouth’s muscles (Roitman et al., 2005). Altogether, these findings indicated that NAc units (mostly from the NAcSh, but also from the NAcC) with inhibitory responses track the motor act of initiating and sustaining food consumption, rather than the sensory properties or value of rewards (Taha and Fields, 2005, 2006). Interestingly, other studies found that individual neurons in the NAc (core+shell) did not only respond to rewarding solutions like sucrose, but also to aversive (bitter) solutions like quinine. Experimenters intraorally infused sucrose and quinine solutions and found that most neurons responded to only one solution. If neurons responded to both tastes, responses were often in the opposite direction. Similar numbers of MSNs responded to sucrose and quinine stimuli and while sucrose predominantly elicited an inhibitory response, quinine predominantly elicited an excitatory one (Roitman et al., 2005). These data indicate that, overall, NAc MSNs track both rewarding and aversive stimuli, which elicit opposite responses (inhibitory for rewarding solutions; excitatory for aversive ones) and engage largely different neural circuits. Overall, the majority of responses, however, are inhibitory responses to food consumption.

One important question relates to which cell types in the NAcSh encode excitation and inhibitory responses during food consumption. Work by O’Connor et al. (2015) used *in vivo* electrophysiology in the medial NAcSh of D1R-cre mice, finding that D1R-MSNs, but not D2R-MSNs, showed a significant inhibition during food consumption. A recent study, however, used *in vivo* single-cell calcium imaging in the medial NAcSh, finding that both D1R-MSNs (identified using Pdyn-cre mice) and D2R-MSNs (identified using Penk-cre mice) showed excitatory and inhibitory responses in the NAcSh during food consumption (Pedersen et al., 2022). 70 and 54% of responsive D1R- and D2R-MSN cells, respectively, showed excitatory responses, while 30 and 46%, respectively, showed inhibitory responses. Inhibitory responses were generally lasting the whole duration of the lick bout. The activity of inhibited neurons started and ended at lick start/end and did not change with reward preference, value, or learning, confirming their role in authorizing feeding (as mentioned above). Neurons with inhibitory responses were also more prevalent in the anterior and medial sections of the NAcSh, confirming the existence of topographical gradients (Pedersen et al., 2022). On the other hand, the activity of excited neurons was acute, and changed with preferred vs. unpreferred rewards, confirming that excitatory responses in MSNs from the medial NAcSh encode the relative value of rewards. For instance, authors found a D2R-MSN cluster projecting to the VP that was specifically responsive to an unpreferred reward (very high sucrose solution) (Pedersen et al., 2022). Moreover, authors found that some D1R-MSNs and D2R-MSNs that showed excitatory responses during reward consumption also showed excitatory responses during cues predicting rewards; however, both these responses did not change with learning (like dopamine does). This suggested that medial NAcSh D1R-MSNs and D2R-MSNs that show excitatory responses do not track reward expectation, but rather reward value and cue salience. Inhibitory responses, on the other hand, track reward consumption and are also found in both D1R-MSNs and D2R-MSNs. In sum, both D1R-MSNs and D2R-MSNs exhibit large and long-lasting inhibitory responses that correlate with food consumption and short-lived excitatory responses that primarily encode reward value.

Several studies also aimed at determining the origin of inhibition and excitation, i.e., which brain regions projecting to the NAcSh drive inhibitory and excitatory responses. How NAcSh MSNs can develop inhibitory responses (‘pauses’) has been studied in detail. Reed et al. (2018) found that excitatory inputs from the vHipp, BLA and PVT arriving into the anterior (but not posterior) NAcSh simultaneously reduce their activity during feeding. This reduction in activity was also sufficient to promote feeding (Reed et al., 2018), altogether indicating that pauses in the activity of vHipp, BLA and PVT inputs authorize feeding by dampening NAcSh activity. Moreover, Vachez et al. (2021) found another source of inhibition controlling food consumption, arising from vArky cells from the VP. Authors showed that vArky cells were active and inhibited both MSNs and interneurons in the NAcSh during food consumption while direct photostimulation of vArky cells promoted food consumption, via NAcSh inhibition. In addition, inhibitory responses in NAcSh MSNs could arise from local interneurons (CINs, PV and SST) or indirectly from VTA GABA projection neurons which target CINs rather than MSNs (Al-Hasani et al., 2021), but future studies are needed to address this. Finally, studies have suggested that reductions in NAc neuron activity necessary for feeding could also result from peptidergic signaling (Urstadt et al., 2013) [also reviewed in detail in Castro and Bruchas (2019)]. On the other hand, excitatory responses in the NAcSh involve dopaminergic inputs – which get activated by rewards, cues that predict rewards and salient stimuli (Berke, 2018), as well as subpopulations of excitatory inputs (e.g., vHipp, BLA, PVT, PFC) – which also exhibit excitatory responses during cues and at the onset of reward consumption (Reed et al., 2018). More studies are needed to decipher the functional role of these excitatory inputs as well as other incoming inputs in how they affect NAcSh activity and in turn behavior.

### 4.2 Direct effect of NAc shell MSNs on food consumption

Since a large proportion of NAcSh MSNs ‘pause’ during food consumption, and NAcSh inactivation increases food consumption (see above), this suggested that the NAcSh acts as a permissive gating signal to authorize feeding, where tonic excitation suppresses, and acute pauses permit feeding. To confirm this hypothesis, Krause et al. (2010) acutely excited NAc subregions during ongoing feeding, finding that NAc excitation in both the NAcC and NAcSh abruptly but reversibly disrupted feeding behavior. Altogether these studies showed that the pause in NAcC /NAcSh units is necessary for the initiation and maintenance of consummatory behaviors. Of note, stimulation of MSNs that led to disruption of licking were more effective in the anterior and medial part of the NAc, with less success rate in the posterior part, confirming the existence of an anterio-posterior gradient (Krause et al., 2010). These findings were later confirmed by O’Connor et al., who determined which cell types and subcircuits could be responsible for how the dorsomedial NAcSh regulates food consumption. They found that photoinhibition of D1R-MSNs in the dorsomedial NAcSh promoted food intake even in sated *ad-libitum*-fed mice while photoinhibition of D1R-MSN (but not D2R-MSN) terminals in the LH rapidly ceased feeding (O’Connor et al., 2015). This suggested that the ability of D1R-MSNs ‘pauses’ to promote food consumption is independent of metabolic need. Moreover, authors found that D1R-MSNs, but not D2R-MSNs, decreased food consumption by inhibiting GABA neurons in the LH, identifying a dorsomedial NAcSh subcircuit that is both necessary and sufficient for food consumption. Of note, NAcSh D1R-MSNs also target glutamatergic neurons in the LH; however while the NAcSh→LH GABA projection is aversive and inhibits feeding, the NAcSh→LH glutamate projection is rewarding (role in feeding not formally determined), indicating the existence of functionally opposing output circuits (Zhou et al., 2022). In later work, D1R-MSN projections to the VTA were also shown to regulate food consumption (Bond et al., 2020), similar to their projections to the LH. This effect likely involves direct inhibition of VTA DA neurons, rather than VTA GABA neurons (Yang et al., 2018; Liu et al., 2022; Zhou et al., 2022). In sum, D1R-MSN projections to LH GABA neurons and (likely) to VTA DA neurons inhibit feeding; when D1R-MSNs pause, this authorizes feeding.

How D1R-MSN and D2R-MSN projections to the VP regulate food consumption is less well understood as more studies have focused on the NAc core in this context [e.g., (Soares-Cunha et al., 2020)]. Studies showed that D1R-MSN projections to the VP pause during food consumption, but the actual impact on how much food is consumed is unclear. Moreover, optogenetic stimulation of D1R-MSN-to-VP projections promoted aversion and reduced opto-paired nose pokes. Since this phenotype is largely similar to that of D1R-MSN to LH GABA projection (O’Connor et al., 2015; Zhou et al., 2022), it is possible that D1R-MSN projections to the VP also drives food consumption; this requires further studies. Moreover, a recent study found that optogenetic activation of NAcSh D2R-MSNs (Penk+) to the VP inhibited food consumption, but only when rewards were preferred, suggesting that the effects of NAcSh-VP D2R-MSNs (Penk+) on food intake depend on reward value (Pedersen et al., 2022). In this study, different populations of NAcSh D2R-MSNs (Penk+) got excited during the consumption of preferred rewards vs. unpreferred rewards, suggesting that one function of the excitatory responses found in the NAcSh is to encode reward value and in turn drive adaptive consumption of preferred rewards vs. unpreferred rewards. How the full range of these NAcSh excitatory responses contribute to food intake behavior still remains relatively unclear and warrants additional studies.

In sum, NAcSh D1R-MSN projections to the VP may, like LH and VTA projections, also inhibit food consumption, but this requires further work. NAcSh D2R-MSN projections to the VP do inhibit food consumption, and this depends on reward value, as the food intake inhibition only occurs when rewards are preferred. Overall, the NAcSh projection circuit is a powerful tri-layered food intake inhibitor, whose activity can rapidly stop feeding, while in turn if its activity pauses, this authorizes feeding via disinhibition of VP, LH, and VTA brain regions.

### 4.3 Circuit- or molecularly-defined subpopulations of NAcSh in the control of feeding and reward

**Figure 3:**
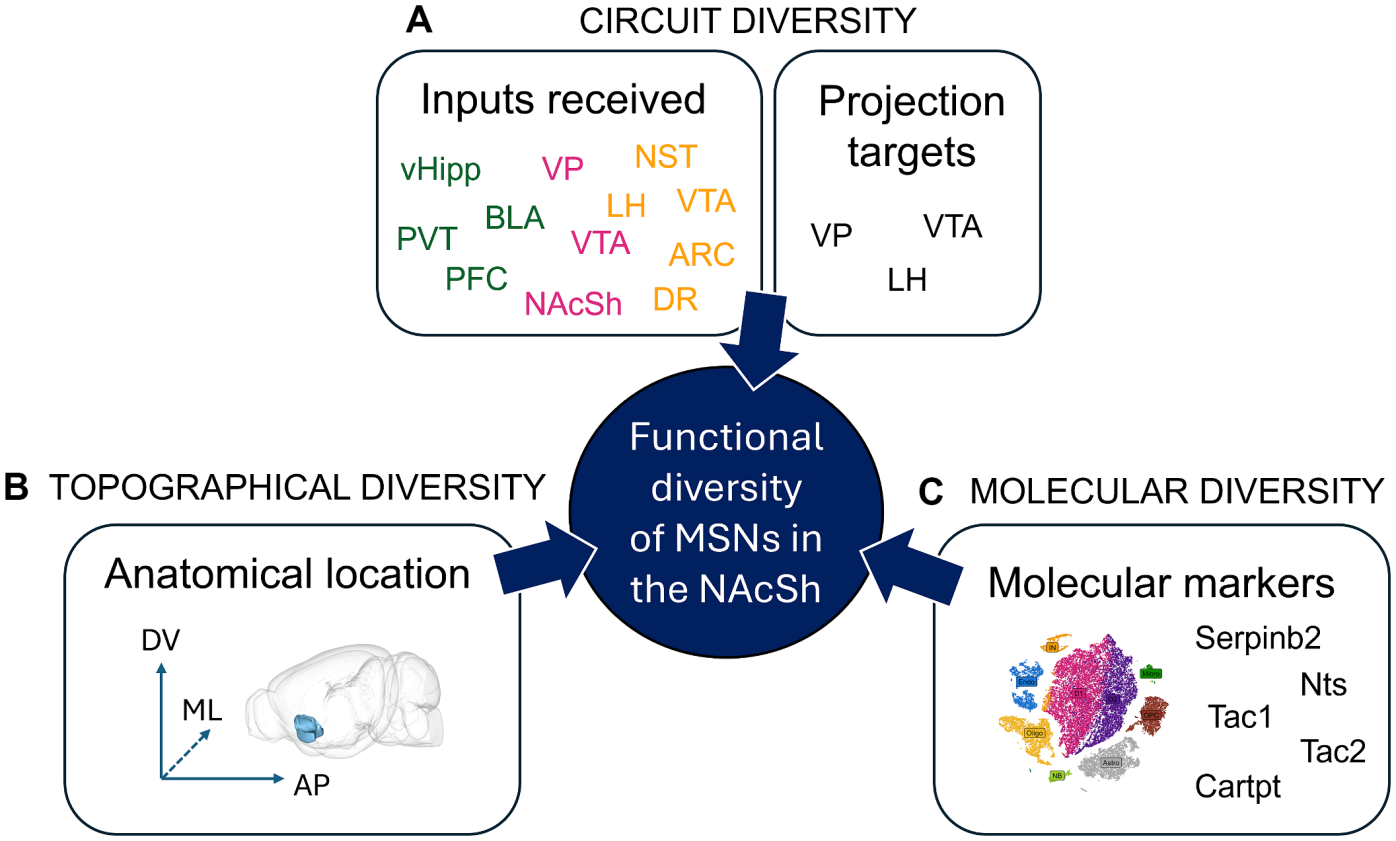
Functional diversity of Nucleus Accumbens Shell projection neurons. Within the NAcSh MSNs expressing D1R or D2R (D1R-MSNs and D2R-MSNs) represent 95% of the cells; they are the output neurons projecting out of the NAcSh. Until recently, it was thought that NAcSh projections neurons can be classified into only two cell types: D1R-MSNs and D2R-MSNs. However, multiple studies have shown the incredible diversity of NAcSh MSNs, where different sets of D1R-MSNs (or D2R-MSNs) harboring distinct circuit, topographical or molecular features can lead to sometimes opposite effects on behavior. **(A)** Circuit diversity defines the fact that various NAcSh MSNs that either receive different inputs (green: excitatory, pink: inhibitory, yellow: neuromodulatory), or project to different output regions, can have differential functional impact, e.g., on behavior. For example, dorsomedial NAcSh MSNs receiving PVT input drive aversion via projections to LH GABA neurons, while dorsomedial NAcSh MSNs receiving BLA input drive reinforcement via VTA GABA and LH glutamate cells (Zhou et al., 2022). **(B)** Topographical diversity defines the fact that MSNs located at different subregions of the NAcSh along the dorso-ventral (DV), medio-lateral (ML) or anterio-posterior (AP) levels can have differential functional impact, e.g., on behavior. For example, while D1R-MSNs (pDyn+) in the ventromedial NAcSh drive aversion, the same cells in the dorsomedial NAcSh drive reinforcement (Al-Hasani et al., 2015). **(C)** Molecular diversity defines the fact that various NAcSh MSNs expressing specific molecular markers can have different functional impact, e.g., on behavior. For example, Serpinb2+ NAcSh cells are primarily found in the dorsomedial NAcSh, comprise a subpopulation of D1R-MSNs, and promote food intake, unlike the canonical dorsomedial NAcSh D1R-MSN which inhibits food intake (Liu et al., 2024). N.B. The 3D brain model in **B**. was obtained from (Lein et al., 2007; Bakker et al., 2015). ARC, Arcuate nucleus of the hypothalamus; BLA, basolateral nucleus of the amygdala; Cartpt, cocaine- and amphetamine-regulated transcript protein; D1R/D2R, dopamine receptor type 1/2; DR, dorsal Raphe nucleus; LH, lateral hypothalamus; MSN, medium spiny neuron; NAcSh, nucleus accumbens shell; NST, nucleus of the solitary tract; Nts, neurotensin; PFC, prefrontal cortex; PVT, paraventricular nucleus of the thalamus; SerpinB2, serpin family B member 2; Tac1, Preprotachykinin-1; Tac2, Neurokinin B; vHipp, ventral Hippocampus; VP, ventral pallidum; VTA, ventral tegmental area.

Understanding how NAcSh MSNs regulate behavior also requires considering their cellular diversity, i.e., the diversity of functions, molecular markers or circuits defined by D1R-MSN or D2R-MSN subpopulations (Figure 3). Indeed, when one records single cell activity of NAcSh cells during behavior, for instance with 2-Photon imaging and Gradient index micro (GRIN) lenses, it is evident that responses are extremely divergent across cells (Pedersen et al., 2022; Chen et al., 2023). Recent studies have also highlighted how otherwise undistinguishable D1R-MSNs can drive sometimes opposite behavioral responses, depending on their anatomical location, cellular inputs, target outputs or molecular markers. The NAcSh is not a uniform structure, but rather a neuroanatomical gradient with “coldspot” and ‘hotspot’ territories along the dorso-ventral, medial-lateral and anterio-posterior axes. Real-time place preference experiments have made a clear demonstration for such spatial heterogeneity – which suggests that similar territories might also exist for food consumption. For instance, Al-Hasani et al. (2015) found that while dynorphin+ D1R-MSNs cells in the ventromedial NAcSh drive real-time aversive behavior, the same cells in the dorsomedial NAcSh drive reinforcement. Yang et al. showed that while lateral NAcSh D1R-MSNs were rewarding, medial NAcSh D1R-MSNs were not (Yang et al., 2018). Richard and Berridge (2011) and Castro and Berridge (2014b) also found differences on the anterio-posterior axis, whereby pharmacological manipulation of anterior vs. posterior NAcSh neurotransmission led to entirely opposite behavioral effects: anterior manipulations led to appetitive responses while posterior manipulations led to defensive responses. Moreover, the NAcSh is not a uniform region simply relaying incoming information via its downstream projections, instead it can be considered a keyboard encompassing multiple and sometimes opposite pathways that can be defined based on the combination of input–output cells. For instance, and as mentioned earlier, dorsomedial NAcSh cells receiving PVT input drive aversion via LH GABA neurons, while those receiving BLA input drive reinforcement via VTA GABA and LH glutamate cells. Furthermore, while NAcSh D1R-MSNs projecting to the VTA drive reward, NAcSh D1R-MSNs projecting to the VP drive aversion (Liu et al., 2022; Zhou et al., 2022). Thus, the NAcSh is composed of intermingled neural circuits that can dictate behavioral outcome via multiple and sometimes opposing sub-circuits. This could represent a tool to fine tune behavioral output (aversion or reward; consumption or not), depending on contextual, sensory, motivational and homeostatic information, all of which are integrated within the NAcSh.

In addition, the molecular diversity of the NAc in general, and the NAcSh in particular, is much greater than originally anticipated, as shown by recent single-cell (sc) RNAseq studies (Stanley et al., 2020; Chen R. et al., 2021). In these studies, although most genes did not follow binary expression rules, several genes were shown to be enriched within regionally defined clusters, including subregions of the NAcSh. For instance, serpin family B member 2 (SerpinB2) + cells were shown to localize in a unique dorsomedial NAcSh D1R-MSN cluster and to promote food intake, unlike canonical dorsomedial NAcSh D1R-MSNs (Liu et al., 2024). Neurokinin (Tac2) + cells labeled a unique D1R-MSN cluster in the NAcSh that prevented cocaine reward, opposite to what canonical D1R-MSNs in this region do (Zhao et al., 2022). Similarly, Preprotachykinin-1 (Tac1) + cells in the NAcSh were found to project primarily to the LH and promote aversion (He et al., 2023). In addition, while lateral NAcSh neurotensin (Nts) + cells promoted reinforcement, CART protein + cells in the medial NAcSh induced aversion (Chen et al., 2023). Mapping specific molecular markers to unique cell types in the NAcSh that drive specific behaviors (e.g., reward vs. aversion, or food consumption vs. food intake inhibition) is tedious. However, it represents a key step to fully dissect the molecular diversity of the NAcSh. It also holds the potential to design novel therapeutic treatments that would target highly specialized circuits within the NAcSh using novel molecular markers.

## 5 Conclusion

In summary, the NAcSh is a unique subregion of the striatum that not only integrates incoming information from sensory and reward-related brain regions but also processes metabolic- and homeostatic-related inputs coming from the hypothalamus, brainstem (and indirectly from the PVT). This unique metabolic input differentiates the NAcSh from the NacC or DS. Unlike the NAcC and DS, the NAcSh also sends dense projections not only to classical striatal output regions, the VP and VTA, but also to the LH, an intermediary brain region directly connected to the mediobasal hypothalamus, i.e., the metabolic center of the brain. Of note, many studies have outlined the existence of sex differences in the organization of the NAcSh, and its relation to behavior; however this field remains at its infancy and requires future work. Important topographical gradients can be found in the NAcSh where more anterior, dorsal and medial regions seem to show the most potent roles in modulating food consumption. Activity in NAcSh D1R-MSNs and D2R-MSNs is multi-faceted with some neurons showing short-lived excitatory responses tracking reward value or other reward-related signals while others show long-lasting inhibitory responses that precisely track the act of consumption. Through its three projections to the VP, LH and VTA, the NAcSh has a powerful impact on feeding behavior with well-established roles in the real-time control of food consumption: activation inhibits feeding while long pauses authorize feeding. This effect can occur even in the absence of metabolic need. NAcSh projections thus have the capacity to promote over- and under-feeding independent of hunger, which makes it a key brain region with potential strong implications in the emergence of altered eating or body weight imbalances like obesity or anorexia nervosa, as shown in several studies (Maldonado-Irizarry and Kelley, 1995; O’Connor et al., 2015; Labouesse et al., 2018; Thoeni et al., 2020). Although less well studied, the NAcSh affects other types of motivated behaviors beyond food consumption, such as food seeking or food liking; how NAcSh projections contributes to these specific behaviors requires future work, however. Of note, the NAcC is also involved in feeding and body weight regulation, potentially acting via similar pathways but partially distinct mechanisms that involve, among other things, the control of motivation and effort (Matikainen-Ankney et al., 2023; Walle et al., 2024). How could this circuit neuroscience knowledge potentially emerge into novel treatment possibilities in the future? Since the NAcSh, NAcC and DS are largely composed of similar cell types, including D1R-MSNs and D2R-MSNs, classical markers like D1R and D2R make it difficult to target unique circuits with sometimes opposing behaviors. Here the advent of modern tracing experiments, single-cell physiology or imaging and scRNAseq studies brings about new hopes, as they could potentially trigger the identification of new molecular markers for unique pathways within subregions of the NAcSh or NAcC, which in turn could have selective impacts on desired behaviors (e.g., consumption reduction).

## Author contributions

A-MM: Conceptualization, Formal analysis, Funding acquisition, Investigation, Methodology, Visualization, Writing – original draft, Writing – review & editing. ML: Conceptualization, Funding acquisition, Project administration, Resources, Supervision, Visualization, Writing – review & editing.
