# Corrigendum: The nucleus accumbens shell: a neural hub at the interface of homeostatic and hedonic feeding

**DOI:** 10.3389/fnins.2024.1531676

**Published:** 2024-12-04

**Authors:** Alina-Mãriuca Marinescu, Marie A. Labouesse

**Affiliations:** ^1^Brain, Wire and Behavior Group, Translational Nutritional Biology Laboratory, Department of Health Sciences and Technology, ETH Zurich, Zurich, Switzerland; ^2^Neuroscience Center Zurich, University of Zurich, ETH Zurich, Zurich, Switzerland

**Keywords:** dopamine, food intake, hedonic feeding, homeostatic feeding, neural circuits, nucleus accumbens, nucleus accumbens shell, reward

In the published article, the reference for “(Liu et al., 2022)” in **Section 4.3**, paragraph 2, and in the legend of Figure 3 was incorrectly written as “Liu, Z., Le, Q., Lv, Y., Chen, X., Cui, J., Zhou, Y., et al. (2022). A distinct D1-MSN subpopulation down-regulates dopamine to promote negative emotional state. Cell Res. 32, 139–156. doi: 10.1038/s41422-021-00588-5”. It should be “Liu, Y., Wang, Y., Zhao, Z. D., Xie, G., Zhang, C., Chen, R., et al. (2024). A subset of dopamine receptor-expressing neurons in the nucleus accumbens controls feeding and energy homeostasis. *Nat. Metab*. 6, 1616–1631. doi: 10.1038/s42255-024-01100-0”.

The authors apologize for this error and state that this does not change the scientific conclusions of the article in any way. The original article has been updated.
